# Image Quality Evaluation of Sanda Sports Video Based on BP Neural Network Perception

**DOI:** 10.1155/2021/5904400

**Published:** 2021-10-27

**Authors:** Kai Fan, Xiaoye Gu

**Affiliations:** Physical Education Department, University of International Business and Economics, Beijing, China

## Abstract

In the special sports camera, there are subframes. A lens is composed of multiple frames. It will be unclear if a frame is cut out. The definition of video screenshots lies in the quality of video. To get clear screenshots, we need to find clear video. The purpose of this paper is to analyze and evaluate the quality of sports video images. Through the semantic analysis and program design of video using computer language, the video images are matched with the data model constructed by research, and the real-time analysis of sports video images is formed, so as to achieve the real-time analysis effect of sports techniques and tactics. In view of the defects of rough image segmentation and high spatial distortion rate in current sports video image evaluation methods, this paper proposes a sports video image evaluation method based on BP neural network perception. The results show that the optimized algorithm can overcome the slow convergence of weights of traditional algorithm and the oscillation in error convergence of variable step size algorithm. The optimized algorithm will significantly reduce the learning error of neural network and the overall error of network quality classification and greatly improve the accuracy of evaluation. Sanda motion video image quality evaluation method based on BP (back propagation) neural network perception has high spatial accuracy, good noise iteration performance, and low spatial distortion rate, so it can accurately evaluate Sanda motion video image quality.

## 1. Introduction

For network evaluation problems, there are often many qualitative factors interspersed and blended in complex evaluation problems, which require people to participate in judgment and decision-making by virtue of experience, knowledge, and wisdom [1]. In the previous decision-making evaluation process, with the participation of people, it is inevitable to be affected by adverse subjective factors such as subjective randomness, thinking uncertainty, and cognitive fuzziness [2]. In order to better promote public fitness and facilitate learning and viewing, sports videos also have a variety of needs for editing, segmentation, and integration. BP (back propagation) neural network technology can use its existing evaluation results to directly evaluate the scheme according to the characteristics of the given new scheme, which can not only reduce human uncertainty and improve the accuracy of evaluation results but also greatly reduce the burden of evaluators [3]. From the perspective of network evaluation, neural network can obtain people's experience, knowledge, and views on the importance of each goal through learning the existing evaluation results. With the continuous development of digital TV technology, video image quality evaluation becomes more and more important. The purpose of this paper is to analyze and evaluate the quality of sports video image, through the use of computer language for video semantic analysis and program design, match the video image with the data model constructed by the research, and form the real-time analysis of sports video image, so as to achieve the real-time analysis effect of sports technology and tactics.

For the traditional video directly played without network transmission in the past, some information will be lost during video compression, which has a certain degree of distortion. Compared with the video without network transmission, the quality of Santa motion video will be damaged not only during compression but also during transmission [4]. In the transmission network of Santa motion video, packet loss, delay, jitter, and other problems will affect the quality of Santa motion video, resulting in video image blur, playback pause, and so on [5]. In the process of image segmentation and noise detection, some threshold parameters need to be optimized to improve the evaluation effect. Due to the complex nonlinear relationship between parameters and evaluation effect, a large number of experiments must be done to find the optimal parameters, which not only waste a lot of time but also have no theoretical basis [6]. Most video quality evaluation algorithms use the image quality evaluation algorithm to obtain the quality score of each frame image and then take the average quality value of all frames as the quality value of the whole video [7]. BP neural network has the ability to approach any nonlinear and multilevel mapping through learning [8]. When it is applied to the prediction of nonlinear system, it cannot be limited by the nonlinear model. Only by fully considering the motion characteristics of objects in the video and judging the motion of objects in the video can we better reflect people's real feelings when watching the video. Therefore, based on the image quality evaluation algorithm based on structural similarity, this paper proposes a frame weighting method based on motion estimation and extends the image quality evaluation algorithm to video quality evaluation algorithm.

At present, most video quality evaluation algorithms take the average image quality of all frames as the quality of the whole video, but the objects in the video have visual motion characteristics, and simply taking the average quality of all frames cannot accurately reflect this characteristic [9]. Video image quality evaluation needs to segment the target object image to ensure the coding integrity of the target object. However, the traditional video image quality evaluation standards cannot search the video content, which has a certain difficulty in image segmentation. Due to simple calculation, PSNR is an important method to evaluate video quality for a long time, but a large number of facts show that its evaluation results are inconsistent with people's real feelings in many cases [10]. Aiming at the existing BP neural network algorithm, this paper proposes an optimization scheme based on variable step BP neural network algorithm and applies it to the quality evaluation of Santa motion video image. In the optimization scheme, different strategies are used to optimize the rising and falling stages of step size. Aiming at the defects of rough image segmentation and high spatial distortion rate in the current sports video image evaluation methods, a sports video image evaluation method based on BP neural network perception is proposed. Experimental results show that this method has high spatial accuracy, good noise iteration performance, and low spatial distortion rate and can accurately evaluate the quality of Santa motion video image.

## 2. Related Work

The existing literature has partially improved the BP neural network. Literature [11] proposed the additional momentum method and the method of dynamically adjusting the learning rate. Literature [12] proposed a method to solve the problem that the network tends to fall into a local minimum. Literature [13] proposed methods such as improving the transfer function, designing a composite error function, and dynamically adjusting different learning rates in layers to improve the network learning rate and anti-interference ability. Literature [14] proposed an SSIM algorithm based on structural similarity based on the structural sensitivity principle of the human visual system to natural images. On this basis, the original algorithm was extended to MSSIM through repeated low-pass filtering and down-sampling of the image so that its performance has been greatly improved. Literature [15] uses wavelet for image analysis, quantifies the observable natural image distortion through two thresholds and extends it to the video quality evaluation, and has achieved good results. Literature [16] directly used the method of planar image quality evaluation in Sanda motion video image quality evaluation. Because this method does not consider the unique distortion types and stereoscopic distortion of stereo images, the accuracy of the evaluation results is low. Literature [17] combines depth information and planar image quality evaluation to evaluate Sanda motion video image quality. Literature [18] divides Sanda motion video image quality evaluation into two parts: depth perception evaluation and left and right viewpoint image evaluation. Literature [19] designed a color and depth stereoscopic video quality evaluation method by extracting the edge information of the depth map and the corresponding color map information.

Because of its simple structure, stable working state, and easy hardware implementation, BP network has been widely used in pattern recognition and classification, system simulation, intelligent fault diagnosis, image processing, function fitting, and optimal prediction. Literature [20] introduces in detail the commonly used objective evaluation methods of image quality without reference. These methods use different fitting formulas, but the visual characteristics of human eyes are very complex, and simple fitting formulas cannot achieve ideal results. Literature [21] uses GAP-RBF and BP network to evaluate the quality of JPEG images. The extracted feature parameters include edge amplitude, edge length, background activity, and background brightness and consider the visual characteristics of human eyes. Literature [22] uses CBP network to achieve objective image quality evaluation of JPEG images. The extracted feature parameters include absolute value, covariance, contrast, and entropy difference. Literature [23] also uses the CBP network to realize the quality evaluation of the MPEG-2 compressed code stream. The characteristic parameters are directly extracted from the compressed code stream, which can realize real-time image quality monitoring. Based on the improved BP neural network algorithm, this paper establishes a Sanda motion video image quality evaluation model so that the model continuously learns the inherent patterns in the training samples. After the training is successful, it can pass the input of various sports indicators and output the evaluation results.

## 3. Materials and Methods

### 3.1. BP Neural Network Technology

The main structural units of artificial neuron are signal input, comprehensive processing, and output, and the intensity of output signal reflects the influence of this unit on adjacent units. The artificial neurons are interconnected to form a network, which is called artificial neural network. The connection mode between neurons is called the connection mode, and the connection degree between neurons is reflected in the artificial neural network by the connection weight. Changing the information processing process and its ability is the process of modifying the network weight.

BP (back propagation) network is a multilayer feedforward network trained by error back propagation algorithm, which is one of the most widely used neural network models at present [24]. At present, there is still no good understanding of the perceptual factors that affect stereoscopic vision in human visual characteristics, so it is a difficult point to study the influence of various perceptual characteristics in stereoscopic images on the image quality of Sanda motion video [16].

**Figure 1:**
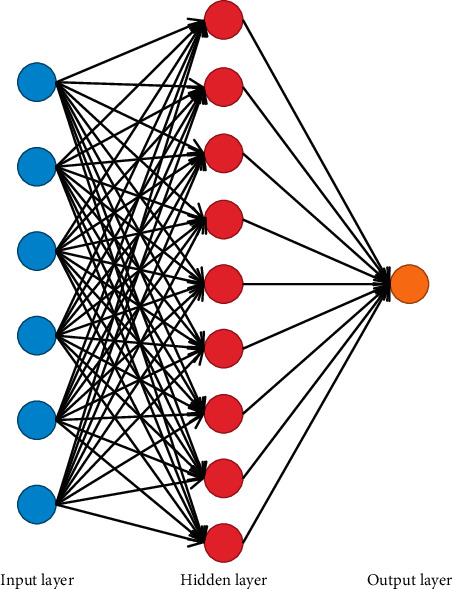
Logical structure of BP neural network.

The BP neural network in this study is 6 inputs, 1 output, and 1 hidden layer. Too many hidden layers will lead to slow training speed and poor convergence effect and are more likely to cause the problem of overlearning, so a three-layer BP neural network is adopted. Experiments show that when the number of hidden nodes is 9, the training effect is the best. The logical structure diagram is shown in Figure 1.

In the process of network training, all samples are brought into the network for training. In the training process, the weights of hidden layers are constantly adjusted through transfer functions. After that, these samples are brought into the network training again and repeated many times so that the weights can be closer to the expected value. In the learning stage of training the network, there are *N* training samples. First, assume that the input and output mode of one of the samples *p* is used to train the network for {*x*^*p*^} and {*t*^*p*^}. The *i*-th neuron of the hidden layer is under the action of sample *p*, which is input as follows:

(1) $$\begin{array}{c} {\text{net}}_{i}^{p}=\sum_{j=1}^{M}\omega_{ij}x_{j}^{p}-b_{i}, \end{array}$$

where *x*_*j*_^*p*^ is the input of the input node *j* when the sample *p* is applied, *w*_*ij*_ is the connection weight between the input layer neuron *j* and the hidden layer neuron *i*, *b*_*i*_ is the threshold value of the hidden layer neuron *i*, and *M* is the input layer. The learning process adjusts the weighting coefficient in the direction that makes the error function *E* decrease the fastest. The weighting coefficient increment formula of any neuron in the output layer and the hidden layer when all samples are applied is

(2) $$\begin{array}{c} W\left(k+1\right)=W\left(k\right)+xite\;dw\;. \end{array}$$

After calculating the new weighted value, substitute it into the next sample. After all the samples are substituted, repeat the above samples to reduce the mean square error of the system and achieve the purpose of learning.

### 3.2. Santa Motion Video Quality Feature Extraction

Sanda sports video quality evaluation requires high real-time performance, so video data should not be processed after video playback, but should be acquired along with video playback. For the characteristic parameters of transmission damage, it is necessary to store each frame of video image in the form of two-dimensional matrix, and the element value in the matrix is the brightness of image pixels [25]. When people make subjective evaluation, they can give the quality of video images without using the original video as a reference, even most video images have never been seen before. This is mainly because people have seen videos of various qualities before. For neural networks, the prerequisite of supervised learning is to have prior knowledge of video quality, that is, to need subjective evaluation value of video quality. In this paper, the neural network model chooses to use one hidden layer, the hidden layer adopts sigmoid transformation function, nine hidden nodes are set, and the output layer has one node, which is the objective evaluation result. Although the output layer chooses linear elements, the hidden layer is nonlinear, so the whole network is also nonlinear. In the process of neural network training, 80% of the video sequences in the data set are used as training samples, and the training sequence of the samples is random.

Feature parameters are used as the input of the system in machine learning and quality prediction, so when choosing feature parameters, it is required that they not only reflect the distortion of the image but also have high consistency with the subjective evaluation value (DMOS). According to this requirement, five characteristic parameters are selected, which are phase consistency (PC), gradient similarity (GS), brightness similarity (LS), contrast similarity (CS), and color similarity (CLS). Adopt phase consistency feature algorithm:

(3) $$\begin{array}{c} \text{PC}\left(x\right)=\frac{\sum_{j}E_{\theta_{i}}\left(x\right)}{\varepsilon +\sum_{n}\sum_{i}A_{n,\beta }\left(x\right)}. \end{array}$$

Among them, *E* (*x*) is the local energy of the image, and *A* (*x*) is the local amplitude of the image. The normalized phase consistency PC is

(4) $$\begin{array}{c} \text{PC}=\frac{2\cdot {\text{PC}}_{1}\cdot {\text{PC}}_{2}+T_{1}}{{\text{PC}}_{1}^{2}+{\text{PC}}_{2}^{2}+T_{1}}. \end{array}$$

Among them, PC_1_ is the phase consistency of the reference image, PC_2_ is the phase consistency of the distorted image, and *T*_1_ is a constant. Gradient is the edge information of the image, and the human eye is more sensitive to the edge information. The image is down-sampled first, and then the Sobel operator is used to calculate the gradient of the image. The gradient information amplitude GM of the image can be defined as

(5) $$\begin{array}{c} \text{GM}=\sqrt{G_{x}^{2}+G_{y}^{2}}. \end{array}$$

Among them, *G*_*x*_ and *G*_*y*_ are the gradient values calculated in the horizontal and vertical directions, respectively. The gradient similarity GS is

(6) $$\begin{array}{c} \text{GS}=\frac{2\cdot {\text{GM}}_{1}\cdot {\text{GM}}_{2}+T_{2}}{{\text{GM}}_{1}^{2}+{\text{GM}}_{2}^{2}+T_{2}}, \end{array}$$

where GM_1_ is the gradient information amplitude of the reference image, GM_2_ is the gradient information amplitude of the distorted image, and *T*_2_ is a constant. The brightness contrast LS calculation formula is

(7) $$\begin{array}{c} \text{LS}=\frac{2\cdot \mu_{1}\cdot \mu_{2}+T_{3}}{\mu_{1}^{2}+\mu_{2}^{2}+T_{3}}, \end{array}$$

where *μ*_1_ is the average value of the reference image block, *μ*_2_ is the average value of the distorted image block, and *T*_3_ is a constant. The calculation formula of contrast similarity CS is

(8) $$\begin{array}{c} \text{CS}=\frac{\sigma_{12}+T_{4}}{\sigma_{1}\cdot \sigma_{2}+T_{4}}, \end{array}$$

where *σ*_1_ is the variance of the original image, *σ*_2_ is the variance of the distorted image, *σ*_12_ is the covariance of the original image and the distorted image, and *T*_4_ is a constant.

Human understanding of images is mainly based on the low-level features of images. Based on physiological and psychological studies, it is found that the image features perceived by human beings are always located at points with high phase consistency. Therefore, phase consistency can be regarded as a simple and effective method to simulate the observation and perception of image features by human visual system, and it is a dimensionless method to reflect the importance of local structural information. For BP neural network, with the training set and input features, it can objectively evaluate the output video quality after training [16]. Then, the parameters of BP neural network determine the similarity between objective evaluation and subjective evaluation.

### 3.3. Sanda Motion Video Quality Evaluation Framework Based on BP Neural Network

**Figure 2:**
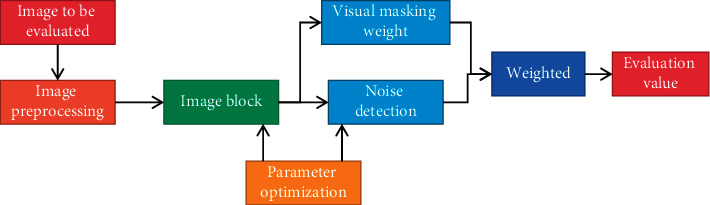
The overall framework of the evaluation system.

It is very important to choose the initial weight when learning BP. In order to make the error as small as possible, it is necessary to choose the initial weight and offset reasonably. When training BP neural network, in order to speed up the training speed and ensure the reliability and stability of the results, the training parameters are set as follows: the training times are 10000 times, the reverse correction step size is 0.05, and the training target is 0.002. Experiments show that this parameter setting can not only ensure the accuracy but also avoid the problem of over-fitting, and the time complexity is very low. When people make subjective evaluation, they can give the quality of video images without using the original image as a reference, even most video images have never been seen before. This is mainly because people have seen images of various qualities before, or these images have similarities in some features. Therefore, for the objective image quality evaluation without reference, the prerequisite is to have prior knowledge of the image quality, and the acquisition of prior knowledge is mainly the classification process of some feature sets of the image. The overall framework of Sanda sports video quality evaluation system based on BP neural network is shown in Figure 2.

In this system, firstly, it is necessary to set the region of interest of human eyes for each selected video sample material and then extract the feature parameters of the region of interest. At the same time, the subjective evaluation results of sample materials are also stored in the database. After that, BP network is trained for the neural network sample database, the characteristic parameters are used as the input of the neural network, and the corresponding subjective evaluation results are used as the expected output. After the neural network is trained, we get the weight of the neural network. For the test material, the same process of region of interest setting and feature extraction is carried out to obtain the feature parameters. Then, the characteristic parameters are used as the input node of the neural network. According to the trained neural network weights, the objective evaluation results can be obtained on the output node of the neural network.

## 4. Result Analysis and Discussion

The evaluation standard is still in accordance with the VQEG (Video Quality Experts Group) inspection standard for objective quality evaluation. The research needs to overcome the shortcomings of slow weight convergence speed of traditional algorithms and error convergence oscillation of variable step size algorithms. Four performance indicators were used to evaluate the corresponding models, namely, PLCC (Pearson linear correlation coefficient), SROCC (Spearman order correlation coefficient), KROCC (Kendall order correlation coefficient), and RMSE (root mean square error). The resolution of high-definition image is 1920 × 1080, and the data amount is about 5 times that of standard-definition image. Moreover, the 16 : 9 display makes subjective evaluators unable to notice all the positions of the whole picture, so only the areas of interest to human eyes are processed in one image. The effectiveness of the five feature parameters selected in the Sanda motion video image quality evaluation model was tested, the number of input feature parameters was sequentially reduced, and the model training was performed. The results are shown in Table 1.

It can be seen that with the decrease of input feature parameters, all the indexes are in a downward trend, which indicates that these five feature parameters are all contributing and effective feature parameters in fusion. At the same time, it can be seen that the characteristic parameters LC and RC contribute the most, which shows that the quality of stereo image is closely related to the distortion degree of left and right viewpoints. The indexes of the last two groups of data are similar, which is mainly because the selected database is symmetrical and distorted, and the quality of the left and right viewpoints of the image pair is similar, so the results are similar.

**Figure 3:**
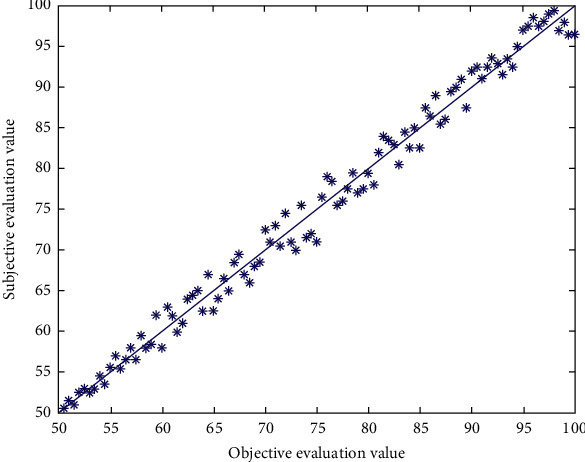
Scatter diagram of image quality evaluation model based on BP neural network.

Figure 3 is a scatter diagram of objective evaluation value and subjective evaluation value of Sanda motion video image quality evaluation model. The more concentrated the scatter points, the better the consistency between objective model and subjective perception. The curve in the figure is the result of nonlinear fitting with five parameters.

**Figure 4:**
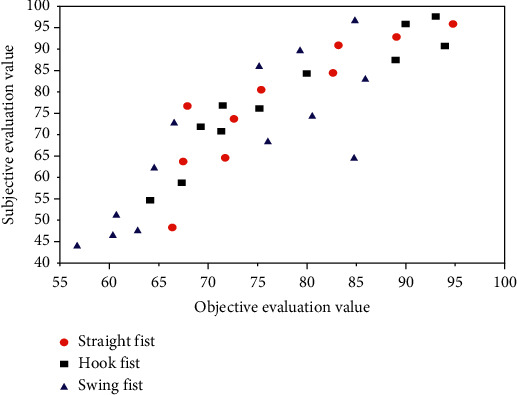
The degree of fit between the subjective evaluation results of the training samples and the objective evaluation results.

**Figure 5:**
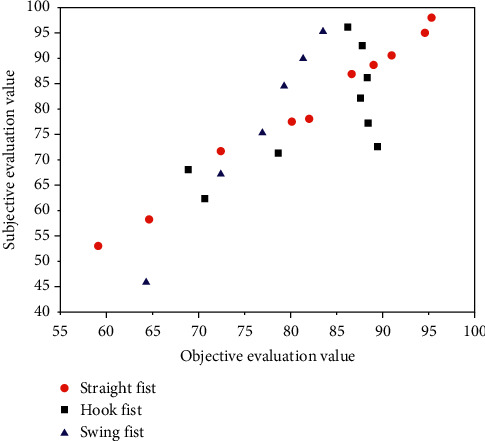
The degree of fit between the subjective evaluation results of the test sample and the objective evaluation results.

The scale of training sample set is extremely important; too few sample sets make little sense to learn; too many sample sets affect the training speed and even lead to failure to converge to the predetermined accuracy. If the block predicted by motion vector in the original video is quite different from the corresponding block in the distorted video, it means that the object in the frame is moving violently. Therefore, the degree of distortion perception of the frame is very small, and the frame is given a smaller weight. Because each index is different from each other, the order of magnitude of each vector in the original sample is very different, which may make neurons in a saturated state, thus losing their learning ability. Figures 4 and 5 respectively show the fitting diagrams of subjective evaluation results and objective evaluation results of training samples and test samples, and the fitting degrees reach 98.45% and 92.08%, respectively.

**Figure 6:**
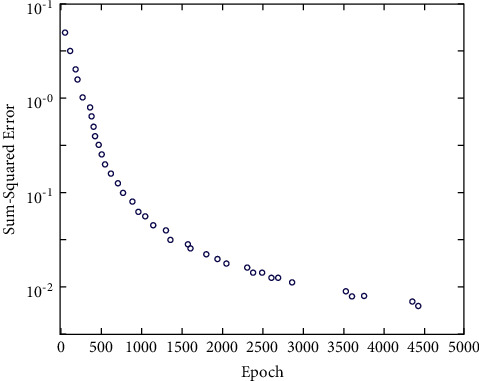
Network training/testing SSE curve.

According to the test results of the test materials, the correlation between subjective evaluation results and objective evaluation results is 92.08%, which has achieved ideal results. Some points in Figure 5 are far from the diagonal, which is mainly because there are not enough materials in the training sample database of neural network, so it is necessary to add various materials to improve the training sample database, so as to get better test results and make the trained network suitable for more different types of test materials. Using the trained network and its generalization ability, the threshold parameters are optimized to improve the evaluation effect of the evaluation method. The trained network optimizes the threshold parameters and generalizes the optimized data according to a large number of networks. The SSE curve of network training/test is shown in Figure 6.

**Figure 7:**
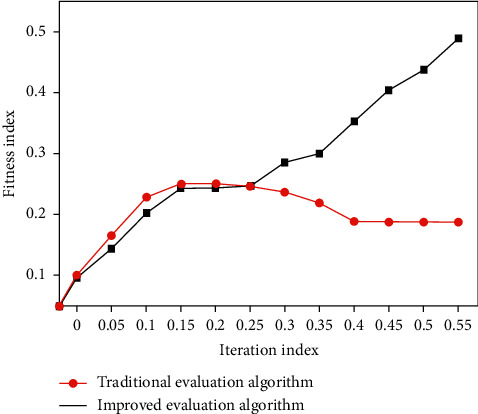
Simulation comparison of image segmentation filtering optimization.

In order to facilitate calculation and prevent some neurons from reaching supersaturation state, the training and test sets are standardized, and the output results after prediction are standardized. In order to make it more intuitive, antistandardization is carried out. After BP neural network optimization, the image segmentation topology is greatly optimized, thus reducing the number of nodes. The simulation comparison of image segmentation topology reliability optimization is shown in Figure 7.

**Figure 8:**
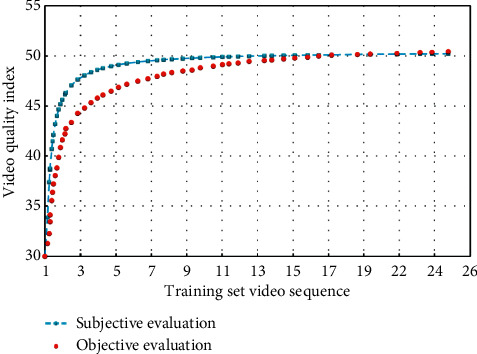
Subjective and objective evaluation values of the experimental training set.

**Figure 9:**
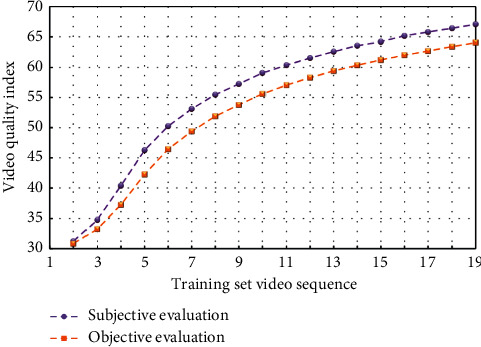
Subjective and objective evaluation values of the experimental test set.

There is little difference in gray level difference between different frames of images in the background, but the gray level difference between different frames of images in the foreground must be larger than the background. According to this property, gray value can be regarded as an important attribute when choosing fuzzy attribute. Figures 8 and 9 show the fitting diagrams of subjective evaluation results and objective evaluation results of training samples and test samples, respectively, with fitting degrees reaching 98.25% and 92.07%. According to the test results of the test materials, the correlation between subjective evaluation results and objective evaluation results is similar, which has achieved ideal results.

Among them, iterative training is stopped when the fitting degree reaches 98.25%, which shows that there is a strong correlation between the selected feature parameters and subjective quality scores, and at the same time, it avoids the over-fitting of training. The fitting degree of the test set is 92.07%, which shows that the quality evaluation result of this model is close to the subjective quality score. In the process of network training, the error limit should be determined in advance according to the actual situation. The selection of error threshold is determined completely according to the convergence speed of the network model and the learning accuracy of specific samples.

## 5. Conclusions

BP neural network is an effective method to study nonlinear and uncertain problems. This model overcomes the shortcomings of multiple regression model and gray model and does not need to determine the mathematical expression form of mathematical model in advance, thus obtaining higher fitting accuracy. This paper mainly introduces the objective evaluation system of Sanda motion video image quality based on BP neural network. In this model, feature parameters which are consistent with subjective evaluation values are used as image feature information. Firstly, BP neural network trained on plane database is used as feature extractor to extract image feature parameters and then combined with absolute disparity map to extract feature parameters. Then, BP neural network is used for feature fusion, and a model is established to evaluate the quality of stereo images. From the network training and test results, it can be seen that the model has a good fitting effect, and the prediction accuracy is also very ideal, which can provide a certain reference for the selection of threshold parameters. Finally, the generalization ability of the network is used to optimize the threshold parameters, which improves the evaluation effect of the evaluation method and saves a lot of test time. The optimized algorithm will greatly reduce the learning error of neural network and the overall error of network quality classification and greatly improve the accuracy of evaluation.

Sanda motion video image quality evaluation model based on BP network can be popularized and applied in many ways. Its input end can be various motion indexes, and its output end can be a comprehensive evaluation result, and it can also be the predicted changes of various input parameters in a short time. Because the sample database of high-definition video sequences is not large enough, it is necessary to continuously add more materials with different damage types to make the network have better generalization ability. In the next step, we will consider extracting the characteristic parameters of distorted images separately and establish an unreferenced Sanda motion video image quality evaluation model to further improve the evaluation performance of the model.

## Figures and Tables

**Table 1:** Validity test results of characteristic parameters.

Characteristics/indicators	PLCC	SROCC	KROCC	RMSE
All features	0.9333	0.9346	0.7601	4.7715
LC + RC + GS + LS	0.9364	0.9314	0.7502	5.2243
LC + RC + GS	0.9282	0.8372	0.6833	6.8196
LC + RC	0.8793	0.8589	0.6653	7.1183
LC	0.8654	0.8566	0.6688	7.2214

## Data Availability

The experimental data used to support the findings of this study are available from the corresponding author upon request.
